# A comparison study of dental pulp stem cells derived from healthy and orthodontically intruded human permanent teeth for mesenchymal stem cell characterisation

**DOI:** 10.1371/journal.pone.0279129

**Published:** 2022-12-27

**Authors:** May Nak Lau, Wijenthiran Kunasekaran, Yue Yuan On, Li Jin Tan, Nurshafiqah Athirah Zaharin, Sarah H. A. Ghani, Sabri Musa, Roziana M. Razi, Gokula Mohan

**Affiliations:** 1 Department of Paediatric Dentistry & Orthodontics, Faculty of Dentistry, Universiti Malaya, Kuala Lumpur, Malaysia; 2 Cytonex Sdn Bhd, Bangsar, Kuala Lumpur, Malaysia; 3 Department of Biochemistry, University of Cambridge, Cambridge, United Kingdom; 4 Department of Cell and Molecular Biology, Faculty of Biotechnology and Biomolecular Sciences, Universiti Putra Malaysia, Serdang, Selangor, Malaysia; 5 Institute of Biological Sciences, Faculty of Science, Universiti Malaya, Kuala Lumpur, Malaysia; Polytechnic University of Marche, ITALY

## Abstract

The objective of this study was to compare the characteristics of Dental Pulp Stem Cells (DPSCs) derived from healthy human permanent teeth with those that were orthodontically-intruded to serve as potential Mesenchymal Stem Cells (MSC). Recruited subjects were treated with orthodontic intrusion on one side of the maxillary first premolar while the opposite side served as the control for a period of six weeks before the dental pulp was extracted. Isolated DPSCs from both the control and intruded samples were analyzed, looking at the morphology, growth kinetics, cell surface marker profile, and multilineage differentiation for MSC characterisation. Our study showed that cells isolated from both groups were able to attach to the cell culture flask, exhibited fibroblast-like morphology under light microscopy, able to differentiate into osteogenic, adipogenic and chondrogenic lineages as well as tested positive for MSCs cell surface markers CD90 and CD105 but negative for haematopoietic cell surface markers CD34 and HLA-DR. Both groups displayed a trend of gradually increasing population doubling time from passage 1 to passage 5. Viable DPSCs from both groups were successfully recovered from their cryopreserved state. In conclusion, DPSCs in the dental pulp of upper premolar not only remained viable after 6 weeks of orthodontic intrusion using fixed appliances but also able to develop into MSCs.

## Introduction

Mesenchymal stem cells (MSCs) are a type of adult stem cells (ASCs) that can be isolated from different sources such as bone marrow, adipose tissue, umbilical cord tissue, and amniotic fluid [1]. They arise from the mesoderm, making it obtainable from different parts of the human body including the pulp tissues in human permanent teeth [2]. MSCs derived from dental pulp tissues are specifically called dental pulp stem cells (DPSCs) [3]. Discovered in 2000, DPSCs are recognized to have the minimum identification criteria of MSCs assigned by the Mesenchymal and Tissue Stem Cell Committee of the International Society for Cellular Therapy (ISCT) [2, 4]. The most significant value of DPSCs compared to other origins of MSCs is the fact that they can be obtained during tooth extraction for tooth impaction and orthodontic treatment where the extracted tooth can be banked for future utilizations [2].

Orthodontics is a specialized field of study in dentistry dealing with the diagnosis and treatment of malocclusion, or teeth misalignment [5]. Statistical reports have shown a rising trend in the uptake of orthodontic treatment in adults worldwide [6–8]. Therefore, tooth banking may be a worthwhile consideration if DPSCs derived from the dental pulp of orthodontically-treated teeth can remain viable for future utilization.

Most orthodontic treatments nowadays include the usage of fixed appliances directly bonded to the teeth to allow three-dimensional control for precise tooth movements such as torqueing, retraction, protraction, intrusion, extrusion, and derotation. Orthodontic intrusion is suspected to be the most damaging to the dental pulp as compared to other orthodontic tooth movements. Hence, it requires the application of the least magnitude of force in order to avoid iatrogenic damage to the dental pulp. It has been found to result in a temporary decrease in pulpal blood flow compared to extrusive, torqueing, and tipping forces [9, 10]. It is also reported that orthodontic intrusion can elicit biological responses in dental pulp ranging from inflammation and vascular degeneration to root resorption and even the rare case of pulp necrosis [11, 12].

Currently, there are limited studies regarding the impact of orthodontic force on dental pulp, with the majority of them are low in quality. In addition, there is inadequate scientific evidence that demonstrates the effect of orthodontic force on dental pulp tissue as reported in recent studies [13, 14]. However, it still remains unknown whether the DPSCs viability can be altered by orthodontic treatment for future clinical benefits. Therefore, the present study incorporated the isolation of DPSCs from dental pulp of extracted from human permanent premolars following a period of orthodontic intrusion and analysing its viability through the fundamental MSCs characterisation tests such as morphology, growth kinetics, cell surface markers analysis, and multilineage differentiation.

## Materials and methods

### Training and calibration

The investigator underwent training in the laboratory prior to study commencement. Calibration for cell counting which is important for isolation, culture, and expansion of DPSCs and growth kinetics was carried out by assessing intraclass correlation coefficient between the investigator and an expert in stem cells studies.

### Ethical approval and participants recruitment

This study was approved by the Medical Ethics Committee, Faculty of Dentistry, Universiti Malaya (Reference No.: DF CD1415/0092(P)).

### Subject recruitment

Subjects were screened for eligibility using inclusion and exclusion criteria. Volunteers requiring extractions of both maxillary first premolars for orthodontic treatment were recruited. Test teeth consisted of orthodontically intruded maxillary first premolars while control teeth comprised healthy maxillary first premolars not bonded with orthodontic bracket and did not undergo any orthodontic force exertion.

Inclusion Criteria:

- New orthodontic patient with no previous orthodontic treatment
- Medically healthy without systemic or genetic diseases
- Require fixed appliances
- Require extractions of both maxillary first premolars
- Both maxillary first premolars are dentally healthy which do not harbour any fractures, caries, or restorations
- Both maxillary first premolars are relatively well-aligned without noticeable rotation or displacement
- Relatively well-aligned upper arch
- No missing teeth on upper arch except third molars

Exclusion Criteria:

- Had previous orthodontic treatment
- Medically ill with systemic or genetic diseases
- Do not require fixed appliances
- Do not require extractions of both maxillary first premolars
- Both maxillary first premolars with current or previous dental problems such as fractures, caries, or restorations
- Both maxillary first premolars are misaligned with noticeable rotation or displacement
- Relatively crowded upper arch
- Missing teeth on upper arch except third molar

### Orthodontic intrusion

Orthodontic bands were cemented onto all first permanent molars and orthodontic brackets were directly bonded onto upper teeth from second premolar to second premolar including the test teeth but excluding the control teeth using pre-adjusted fixed appliances with MBT prescription and 0.022” x 0.028” slot (Unitek^TM^ Gemini Metal Brackets, Unitek Information Technologies, Sherman Oaks, California, USA). Orthodontic bracket was bonded occlusally on the test tooth with a standardised distance of 3 mm from the archwire to the bracket slot. Orthodontic force in an intrusive direction was applied to the test teeth using 0.016” heat-activated Nickel Titanium archwire (Nitinol Heat-Activated, Unitek Information Technologies, Sherman Oaks, California, USA). The intrusion force applied was approximately 50 g of force measured by a gauge (Correx Federwaage 10–100 g Dial-type Stress and Tension Gauge, Haag-Streit, Berg, Switzerland). Both control and test teeth were extracted after 6 weeks.

### Sample collection

Freshly extracted test teeth and control teeth were immediately kept in normal saline (RinsCap NS, Ain Medicare, Kelantan, MY) before the cutting procedure which was commenced immediately following extraction within 2 hours to extirpate the dental pulp [15]. Extracted teeth were disinfected with povidone iodine (Unidon, Unimed Sdn. Bhd., Kuala Lumpur, MY) before cutting. The coronal part of the teeth near the cementoenamel junction was cut without exposing the dental pulp under copious irrigation of sterile normal saline (RinsCap NS, Ain Medicare, Kelantan, MY). The tooth was broken into half at the cementoenamel junction to expose the pulp chamber. The exposed dental pulp was extirpated and placed into transporting media containing Dulbecco’s Modified Eagle Medium KnockOut (DMEM-KO) cell culture media (Gibco Inc., Billings, Montana, USA).

### Isolation and primary culture of DPSCs

Each dental pulp tissue was washed three times with washing buffer solution containing Dulbecco’s Phosphate Buffered Saline (DPBS -/-) (Gibco Inc., Billings, Montana, USA), 5% Penicillin Streptomycin (Pen Strep, Gibco Inc., Montana, USA) and 5% Antibiotic-Antimycotic (Gibco Inc., Billings, Montana, USA) before being treated with 0.5 ml of 3 mg/ml Collagenase Type 1 solution (Gibco Inc., Billings, Montana, USA). The tissue was then minced into smaller fragments and incubated for 30 minutes at 37°C. The tissue was then transferred into growth media containing DMEM-KO cell culture media, 10% Fetal Bovine Serum (FBS) (Hyclone Laboratories Inc., Logan, Utah, USA), 1% Glutamax (Gibco Inc., Billings, Montana, USA), and 0.5% Penicillin Streptomycin. The sample was centrifuged for 6 minutes at 1200 rcf. The cell pellet was gently resuspended with 1 ml of growth media. Following that, the cells were seeded in T25 cell culture flasks (BD Bioscience, Franklin Lakes, New Jersey, USA) with the growth media and incubated in a humidified atmosphere of 95% air and 5% CO_2_ at 37°C. Two-thirds of the growth media was replaced every 3 days. When the primary culture became subconfluent, the cells were collected by trypsinisation using 2 ml of 0.05% of Trypsin with ethylenediaminetetraacetic acid (Trypsin-EDTA) (Gibco Inc., Billings, Montana, USA) and processed for subsequent passages.

### Cell morphology

Cell morphology was observed, and images were taken using an inverted microscope (DMI3000 B, Leica Camera AG, Wetzlar, DE) at day 1 and every 3 days subsequently before replacing the growth media until the culture became subconfluent.

### Expansion and cryopreservation

For cell subculture, the media was discarded, and the cell culture flask was rinsed with 5 ml of DPBS -/-, detached by 2 ml of 0.05% of Trypsin-EDTA, and incubated at 37°C for 5 minutes before being neutralized with 8 ml of growth media. The solution was centrifuged at 1200 rcf for 6 minutes, and the cell pellet was resuspended with 2 ml of growth media. An aliquot of the growth media was used to determine the total living cell count by Trypan Blue dye exclusion method (Gibco Inc., Billings, Montana, USA) and counted using haemocytometer (Paul Marienfeld GmbH & Co. KG, Lauda-Königshofen, DE). After cell counting at each passage, the cells were seeded into three new T25 cell culture flasks with a cell-seeding density of 1500 cells/cm^2^ and incubated in a humidified atmosphere of 95% air and 5% CO_2_ at 37°C. The growth media was replaced every 3 days until the cell culture became subconfluent for subsequent subcultures. The cells were expanded until the working passage required for each experiment was achieved and the remaining cells were frozen for cryopreservation using freezing media containing 10 ml dimethyl sulfoxide (Sigma-Aldrich, St. Louis, Missouri, USA) and 90 ml of FBS in a cryovial tube which was then stored in liquid nitrogen.

### Growth kinetics

The growth kinetics of the DPSCs were analysed by calculating the population doubling time (PDT) [15]. A total of 1500 cell/cm^2^ of cells were cultured in a T25 cell culture flask with three replicates for each passage. The time taken for each passage to become subconfluent and the total cell count were recorded for five passages.

### Cell surface markers analysis using flow cytometry

Cell surface markers expression were studied at passage 3 using BD FACSCalibur (BD Bioscience, New Jersey, USA). Once subconfluent, the cells were harvested with 0.05% Trypsin-EDTA and resuspended in 10 ml of DPBS -/-. The cell suspension was centrifuged at 1200 rcf for 6 minutes. The cell pellet was resuspended in 10 ml of DPBS -/-. Cell counting was performed to determine the total cell count and to transfer 1 x 10^6^ cells into 12 round bottom tubes. The cells were then centrifuged at 1200 rcf for 12 minutes. The supernatant was removed and the pellet was again resuspended with 100 μl of DPBS -/-. The cells in each tube were added with antibodies for markers as shown in Table 1 and incubated in the dark by covering the tubes with aluminium foil for 15 minutes at room temperature. An additional 2 ml of DPBS -/- was then added into each tube prior to spinning it at 300 rcf for 5 minutes at room temperature. The supernatant was removed and the cells were resuspended with 1 ml of 4% paraformaldehyde. The tubes were then covered with aluminium foil and placed on ice. At least 10,000 events were acquired on a BD FACSCalibur (BD Bioscience, New Jersey, USA).

**Table 1:** List of antibodies used for cell surface markers analysis.

Cell surface marker antibodies	Volume (μl)
CD34 PE	20
CD90 FITC	20
CD105 APC	5
ANTI HLA-DR PERCP-CY5.5	20

### Multilineage differentiation

All samples were plated at a density of 1,500 cells/cm^2^ in a 6-well plate with growth media at passage 3 and incubated in a humidified atmosphere of 95% air and 5% CO_2_ at 37°C. Once becoming subconfluent, the media was discarded and the wells were washed twice with 5 ml DPBS -/- prior to the addition of different differentiation media. Three wells served as control with no induction of differentiation and the other three as experimental wells. The cells were differentiated into osteogenic, adipogenic, and chondrogenic lineages using Osteogenesis Differentiation Medium (ODM), Adipogenesis Differentiation Medium (ADM), and Chondrogenesis Differentiation Medium (CDM), respectively. The cells were then examined by using a light microscope.

### Statistical analysis

Data was analysed using Statistical Package for the Social Sciences (SPSS) for Windows version 23.0 (SPSS Inc., Chicago, Illinois, USA). Intraclass correlation coefficient was performed for inter-rater agreement between the investigator and an expert in stem cells studies for cell counting. Descriptive data were presented as mean and standard deviation (mean ± SD).

## Results

### Orthodontic intrusion

All teeth from the control (N = 20) and test (N = 20) groups were successfully intruded fully (3mm) within 6 weeks.

### Inter-rater agreement

The inter-rater agreement analysis for six samples for training showed that there was an almost perfect agreement (intraclass correlation coefficient = 0.98) between the investigators and the experts on the isolation and culture of DPSCs.

### Sample profile and isolation success rate

A total of 20 control teeth and 20 test teeth were recruited. Only 2 control teeth and 1 test tooth were successfully expanded without contamination to passage 5 for analysis of growth kinetics, cell surface markers, and multilineage differentiation.

Out of 20 control samples, 20% (4/20) of them showed cell growth while 55% (11/20) showed no cell growth. Of the 20 intruded samples, 15% (3/20) showed cell growth while 60% (12/20) showed no growth. The remaining 25% (5/20) of control and intruded samples, respectively, were contaminated during the primary culture. Out of 7 samples that showed cell growth, 57.1% (4/7) of them were contaminated at passage 2, while the remaining 42.9% (3/7) were successfully expanded to passage 5 (66.7% (2/3) of control samples and 33.3% (1/3) of intruded sample).

### Morphology

**Fig 1:**
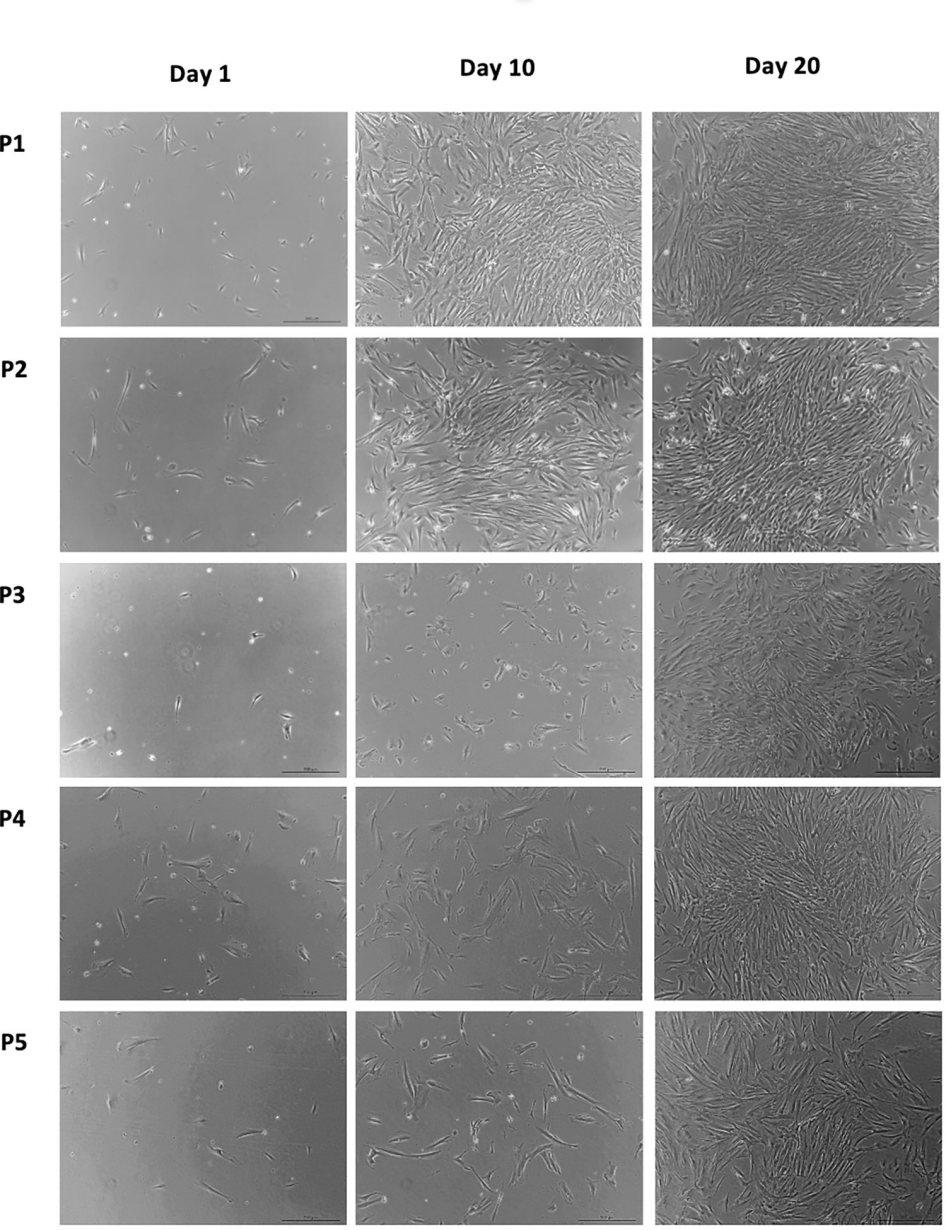
Photographs of cells from control sample on day 1, day 10, and day 20 for all 5 passages at 5x magnification (2 mm = 200 μm).

**Fig 2:**
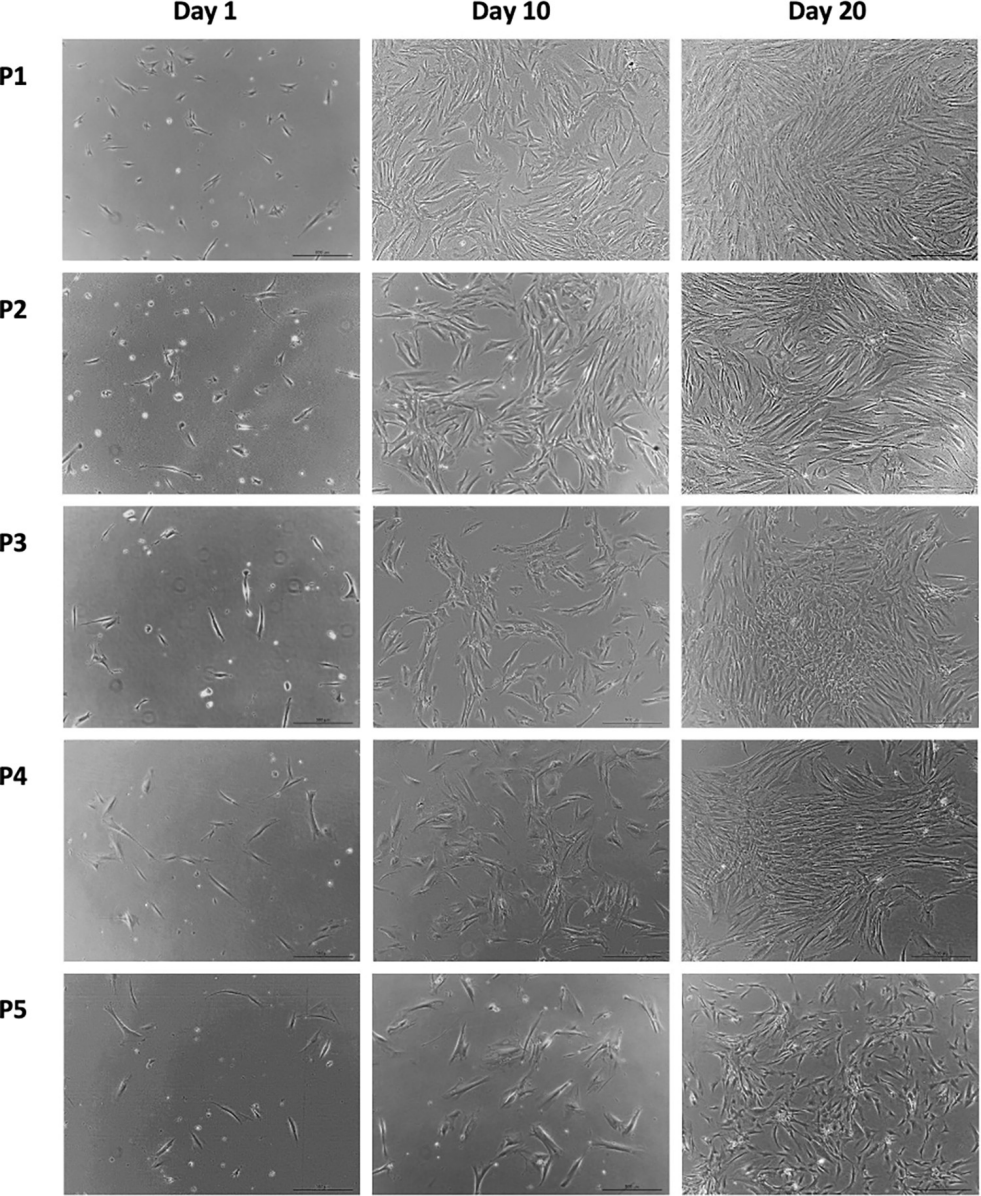
Photographs of cells from test sample on day 1, day 10, and day 20 for all 5 passages at 5x magnification (2 mm = 200 μm).

As shown in Figs 1 and 2, the cells isolated from both control and test groups showed a fibroblast-like, elongated, spindle-shaped morphology when examined under a light microscope. At passage 0, all samples with a successful growth from both control and test teeth began to grow after approximately 14 to 28 days and reached a monolayer of confluence after approximately 42 to 70 days. On subsequent passages, 21 days were taken by the cells to proliferate and reach a monolayer of confluence.

Cells from both groups were able to attach to the plastic cell culture flask. On day 0, cells seeded in the cell culture flask were round in shape and floating in the growth media. On day 1, the stellate shape of cells was apparent and became attached to the plastic cell culture flask as observed using a light microscope.

As the cells were cultured from passage 1 to passage 5, the proliferation rate for both control and test groups were decreased, with the total number of cells notably less through the passages at each time point (day 1, day 10, and day 20).

### Growth kinetics

**Fig 3:**
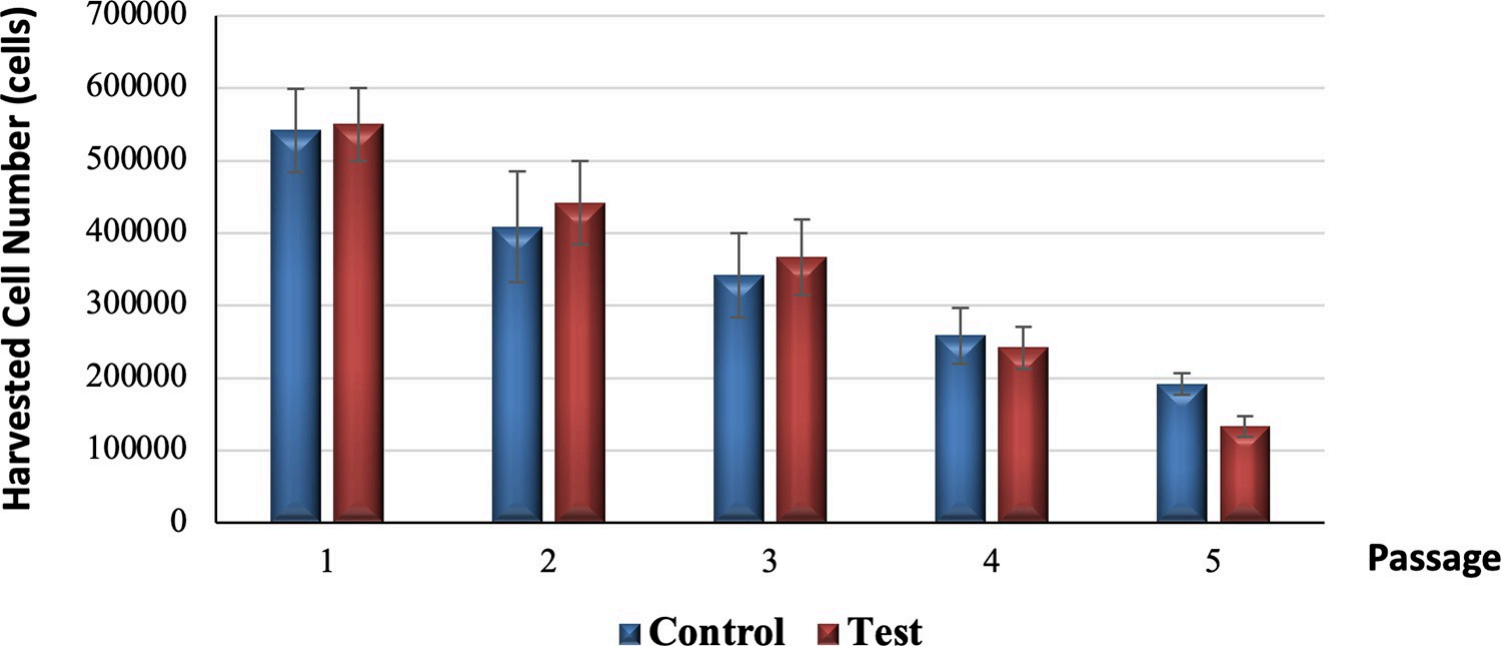
Harvested cell numbers of all five passages between control and test groups.

Fig 3 illustrates the number of cells harvested at each passage from passage 1 to passage 5 for both control and test groups. The number of cells harvested from the test tooth were slightly higher compared to the control tooth in passage 1 (test tooth = 550,000 (S.D. 50,000) cells, control tooth = 541,667 (S.D. 57,735) cells, passage 2 (test tooth = 441,667 (S.D. 57,735) cells, control tooth = 408,333 (S.D. 76,376) cells and passage 3 (test tooth = 366,667 (S.D. 52,042) cells, control tooth = 341,667 (S.D. 57,735) cells by 1.54% (8,333 cells), 8.16% (33,333 cells), and 7.32% (25,000 cells), respectively. In contrast, the number of cells harvested from the test tooth were slightly lower than the control tooth in passage 4 (test tooth = 241,667 (S.D. 28,868) cells, control tooth = 258,333 (S.D. 38,188) cells and passage 5 (test tooth = 133,333 (S.D. 14,434) cells, control tooth = 191,667 (S.D. 14,434) cells by 6.45% (16,667 cells) and 30.43% (58,333 cells) respectively.

**Fig 4:**
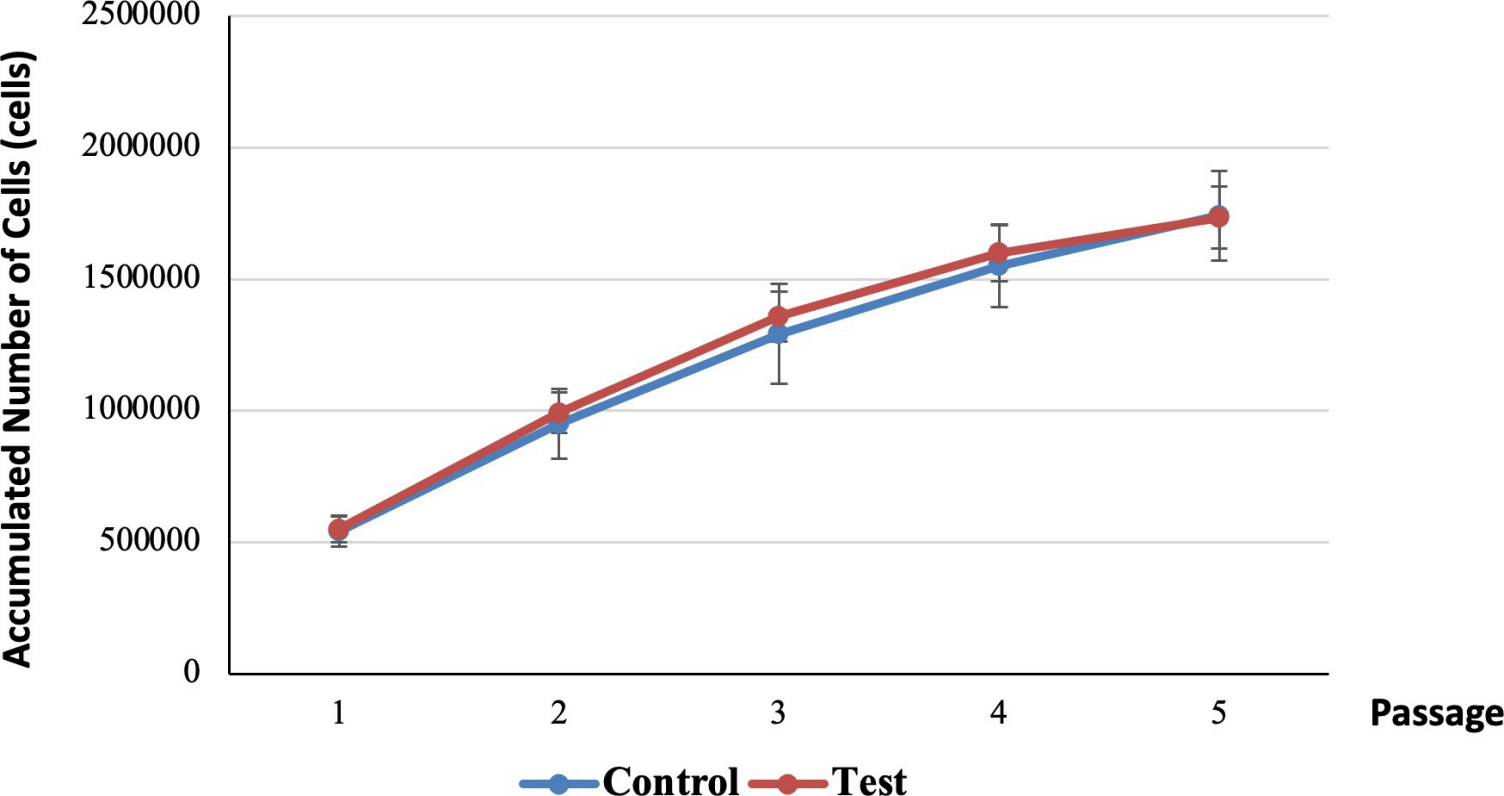
Growth curves of control and test groups up to passage 5.

Further analysis regarding the accumulated number of cells harvested at each passage for both control and test groups is shown in Fig 4. The accumulated number of cells harvested for the test tooth was slightly higher than the control tooth in passage 1 (test tooth = 550,000 (S.D. 50,000) cells, control tooth = 541,667 (S.D. 57,735) cells), passage 2 (test tooth = 991,667 (S.D. 76,376) cells, control tooth = 950,000 (S.D. 132,288) cells), passage 3 (test tooth = 1,358,333 (S.D. 94,648) cells, control tooth = 1,291,667 (S.D. 189,297) cells) and passage 4 (test tooth = 1,600,000 (S.D. 108,972) cells, control tooth = 1,550,000 (S.D. 156,125) cells by 1.54% (8,333 cells), 7.69% (41,667 cells), 5.16% (66,667 cells), and 3.23% (50,000 cells), respectively. On the contrary, the accumulated number of cells harvested for test group (1,733,333 (S.D. 118,145.39) cells) was slightly lower compared to the control group (1,741,667 (S.D. 170,171) cells) in passage 5 by 0.48% (8,333 cells).

**Fig 5:**
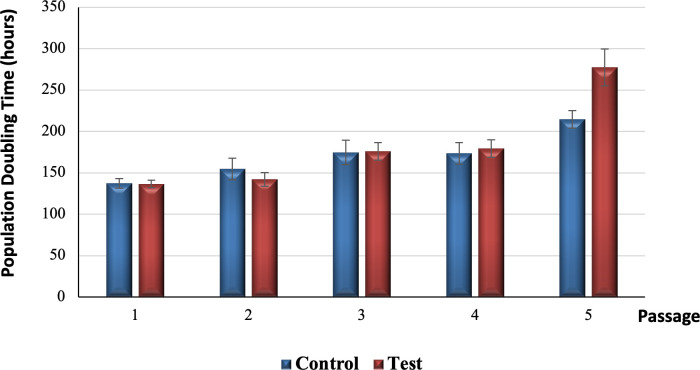
Population doubling time of control and test groups from passage 1 to passage 5.

Fig 5 demonstrates the increasing trend of population doubling time (PDT) for control and test groups from passage 1 to passage 5. For passages 1 to 4, both control and test groups recorded a relatively similar proliferation rate, with the control group having a slightly faster PDT compared to the test group in passage 1 (test group = 136.52 (S.D. 4.65) hours, control group = 137.42 (S.D. 5.82) hours) and passage 2 (test group = 142.30 (S.D. 8.16) hours, control group = 154.79 (S.D. 13.14) hours) by 0.65% (0.9 hours) and 8.78% (12.49 hours), respectively. Meanwhile, the test group recorded a slightly faster PDT compared to the control group in passage 3 (test group = 176.03 (S.D. 10.48) hours, control group = 174.79 (S.D. 14.93) hours) and passage 4 (test group = 179.46 (S.D. 10.80) hours, control group = 173.69 (S.D. 12.93) hours) by 0.71% (1.24 hours) and 3.32% (5.77 hours), respectively. A similar pattern was observed in passage 5 as the test group (277.44 (S.D. 22.03) hours) had a faster PDT compared to the control group (214.72 (S.D. 10.44) hours) with a notably higher PDT (29.21%; 62.72 hours).

### Cell surface markers analysis using flow cytometry

**Fig 6:**
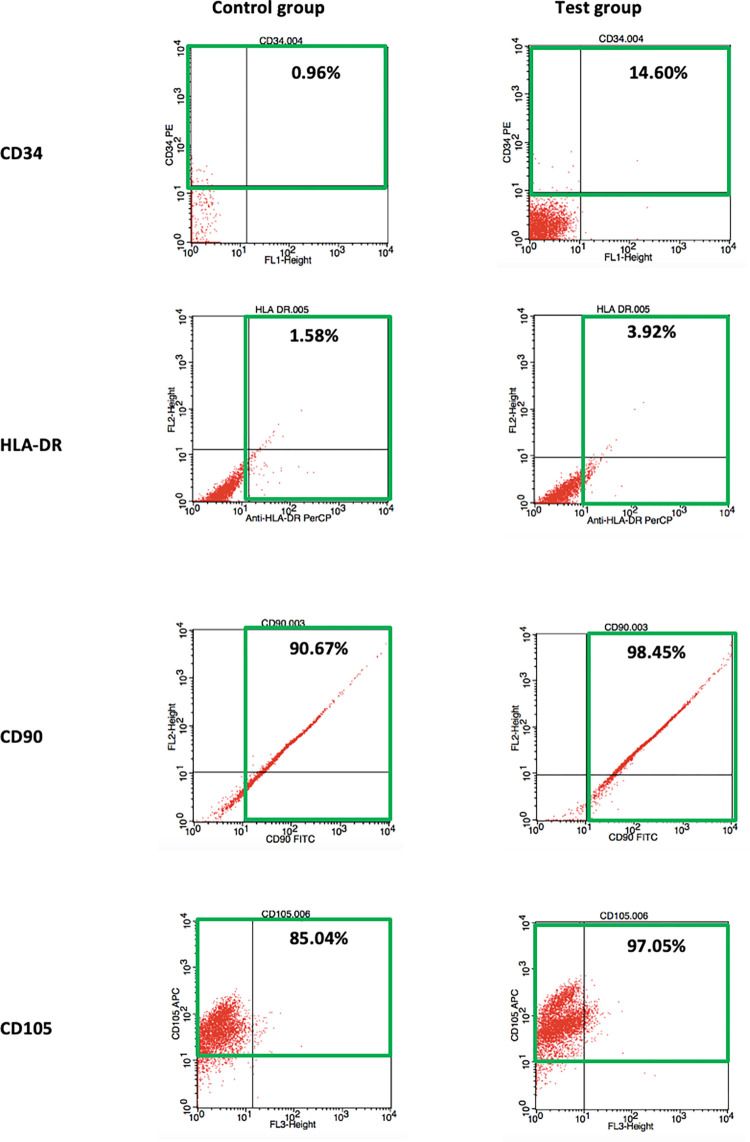
The expression of cell surface markers CD34, HLA-DR, CD90, and CD105 for the control and test groups (positive finding: Within highlighted green box).

Table 2 displays the percentages of cell surface markers including CD34, HLA-DR, CD90 and CD105 on the cells for control and test groups. The cells from control and test groups did express a lesser percentage of haematopoietic markers such as CD34 (test group = 14.6%, control group = 0.96%) and HLA-DR (test group = 3.92%, control group = 1.58%). On the other hand, the cells isolated from control and test groups expressed a higher percentage for MSCs markers of CD90 (test group = 98.45%, control group = 90.67%) and CD105 (test group = 97.05%, control group = 85.04%). The expression of cell surface markers CD34, HLA-DR, CD90, and CD105 for the control and test groups is illustrated in Fig 6.

**Table 2:** The percentages of cell surface markers CD34, HLA-DR, CD90, and CD105 on the cells from control and test groups.

	Control group	Test group
**CD34**	0.96%	14.60%
**HLA-DR**	1.58%	3.92%
**CD90**	90.67%	98.45%
**CD105**	85.04%	97.05%

### Multilineage differentiation

**Fig 7:**
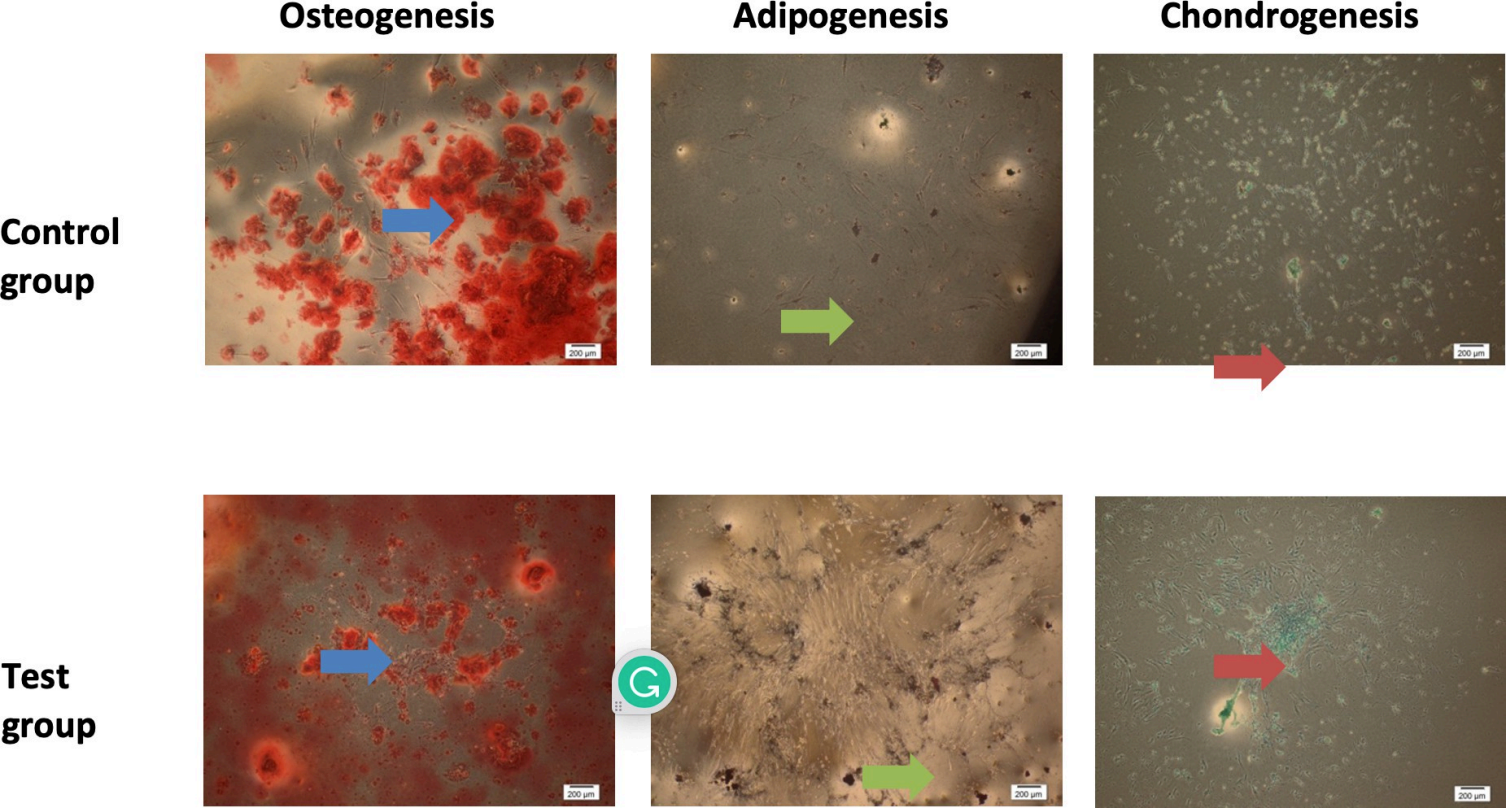
Multilineage differentiation assays (osteogenic, adipogenic, and chondrogenic) of the cells from the control and test groups at 5x magnification (2 mm = 200 μm) (blue arrow: Calcium stained in red; green arrow: Lipid droplets stained in red; red arrow: Proteoglycan stained in blue).

The results obtained from the multilineage differentiation test were similar between control and test groups. DPSCs from both groups were able to differentiate into osteogenic, adipogenic and chondrogenic lineages under appropriate induction media. The presence of red coloured clumps in osteogenic cells indicated the formation of calcium, while the presence of red coloured lipid droplets was observed in adipogenic cells. The formation of proteoglycan stained in blue colour was evident in the chondrogenic cells (Fig 7).

### Cryopreservation and recovery of DPSCs from the cryopreserved state

DPSCs were successfully cryopreserved and viable DPSCs from both control and test teeth were successfully recovered from their cryopreserved state. Observations with light microscope showed the same fibroblast-like morphology and they were able to grow until subconfluent for subsequent tests.

## Discussion

The result of the inter-rater agreement on cell counting was excellent. Since it was not possible to obtain sample replications with the exact number of living cells for cell counting after a washout period, the intra-rater reliability test was not attainable. Cell counting consistency is crucial for cell expansion procedures alongside with the analysis of growth kinetics. The investigators acquired extensive and thorough training from the expert, especially on pulp extirpation, isolation and culture of stem cells, and all biological tests performed in this study. In addition to that, each sample used at every passage was accompanied by a series of opinions and insights from the expert to ensure 90% of confluency before subculture.

Orthodontic intrusion was achieved by applying an intrusive force of about 50 g using 0.016” heat-activated Nickel Titanium archwire in this study. 0.016” Nickel Titanium archwire was selected because it is commonly available in the clinic and commonly used as the initial archwire for a relatively well aligned arch yet large enough to exert a potentially higher force compared to other Nickel Titanium archwires of smaller dimensions such as 0.012" or 0.014" Nickel Titanium archwire. Heat-activated type of Nickel Titanium archwire was chosen for this study because it generates lighter forces over greater deflection plateaus compared to the traditional Nickel Titanium archwire which simulates the common orthodontic clinical practice of using a lighter force for tooth movement [16]. The common intrusive force recommended ranges from 10 to 20 g but it is based on expert opinion instead of evidence. An intrusive force of 40 g and 80 g have been attempted and the increase in the intrusive force did not show any significant increase of intrusion rate [17]. In the present study, orthodontic bracket was bonded occlusally on the test tooth with a standardised distance of 3 mm from the archwire to the bracket slot which is the maximum distance permissible for a maxillary premolar. The force was measured using a gauge (Correx Federwaage 10–100 g Dial-type Stress and Tension Gauge, Haag-Streit, Berg, Switzerland) and it was recorded that the intrusive force exerted on the test tooth was about 50 g.

Regardless of the allocation of treatment, this study had a relatively low isolation success rate. This finding was inconsistent with the previous studies which reported a higher isolation success rate of DPSCs ranging from 77.5% to 96.8% [18–20]. The low isolation success rate in this study might be due to the sample contaminations following insufficient laboratory equipment for tooth cutting and pulp extirpation. In this study, tooth-cutting and pulp extirpation were performed in the Postgraduate Clinic where ultrasonic scaling was frequently performed. Bacterial aerosol contamination due to ultrasonic scaling might be one of the possible factors that contributed to the low isolation success rate of DPSCs in this study [21, 22]. In addition, environmental contaminants may also be present due to the recirculation of bacterial aerosols by the centralised air conditioning system [23, 24]. Our pilot test showed that disinfection of the extracted teeth before cutting and pulp extirpation by povidone iodine has significantly improved the isolation success rate from 20% to 30%. Furthermore, the premolars that contain a lesser amount of pulp tissue used in the present study may have contributed to the lower isolation success rate of DPSCs compared to the third molars. Besides that, the narrow root canal and slender pulp tissue in the premolars did lead to difficulty in pulp extirpation. This is in agreement with Pereira et al. that pulpectomy, a dental procedure to remove all the pulp from the crown and roots of a tooth in one piece is not possible to be performed due to the narrowness in the root canals [25].

The findings of this study described that DPSCs in the dental pulp of upper premolars were viable after being orthodontically intruded for 6 weeks using fixed appliances. Aside from the lack of high-quality studies which led to inadequate evidence of relationship between orthodontic forces and dental pulp, this was in agreement with previous studies which reported both viability and functions of DPSCs were preserved after an orthodontic intrusion throughout the study period [13, 14].

The cells from both control and intruded premolars fulfilled the first criteria of MSCs as they were able to attach to the cell culture flask and exhibited a fibroblast-like, spindle-shaped morphology. There was no difference observed in the appearance of the cells isolated from the control and intruded premolars under the light microscope. Since DPSCs are of mesodermal origin, markers used to identify MSCs can also be used for the identification of DPSCs [26]. Besides, a lack of expression of haematopoietic markers is also one of the criteria for MSCs [4]. The present study confirmed that the cells derived from both the control and test groups displayed high expression for MSCs markers (CD90 and CD105), which was in accordance with an earlier research study by Tatsuhiro et al. [27]. On the other hand, the samples from both control and intruded premolars were lack of haematopoietic markers (CD34 and HLA-DR). This finding was in line with past investigations [27–29]. The expression of 14.6% cells for the CD34 marker found in the test group suggests that there were increased primitive haematopoietic progenitors in the dental pulp following orthodontic intrusion, likely due to changes such as reduction of pulpal blood flow caused by compression on the apical blood vessels. The study also highlighted the ability of the cells derived from both control and intruded premolars to differentiate into osteogenic, adipogenic, and chondrogenic lineages, which was in line with numerous studies conducted worldwide [30–33]. The detection of calcium deposits, lipid droplets, and proteoglycan in the cells isolated from both control and test groups confirmed the presence of osteoblasts, adipocytes, and chondroblasts, respectively. All the findings as discussed above indicated that the cells isolated from both groups in this study have fulfilled the criteria; (1) adherence to plastic, (2) specific surface antigen expression, and (3) multipotent differentiation potential, from the International Society for Cellular Therapy (ISCT) for MSCs definition [4].

Population Doubling time (PDT) was used to determine the proliferation rate of DPSCs with respect to the initial seeding density and time required for stem cells to become subconfluent. To date, optimal initial plating density remains unestablished. Nevertheless, replacing FBS with human serum such as human platelet lysate or reducing the concentration of FBS with the addition of insulin, transferrin, and sodium selenite supplement (ITS supplement) has been proven to increase population doubling significantly [18, 34, 35]. The PDT data of DPSCs is not frequently reported so a direct comparison of the obtained results with previous data is not possible.

### Limitations

Although the results obtained in this study are novel and the objectives of the study were successfully achieved, there are some constraints associated with this study that must be considered for future investigations. This study was limited to a small number of samples (i.e., DPSCs isolates) with a shorter duration of orthodontic intrusion for test premolars (i.e., 6 weeks) prior to extraction which led to a low isolation success rate and contribute to a low number of samples following the subsequent biological tests.

## Conclusion

In conclusion, viable DPSCs were successfully isolated from human permanent premolar subjected to 6 weeks of orthodontic intrusion. The findings of this study suggest that both DPSCs from healthy and orthodontically intruded human permanent premolars fulfilled the criteria of MSCs definition.
